# PLA-Based Composite Panels Prepared via Multi-Material Fused Filament Fabrication and Associated Investigation of Process Parameters on Flexural Properties of the Fabricated Composite

**DOI:** 10.3390/polym16010109

**Published:** 2023-12-29

**Authors:** Zhaogui Wang, Lihan Wang, Feng Tang, Chengyang Shen

**Affiliations:** 1 Department of Mechanical Engineering, Naval Architecture and Ocean Engineering College, Dalian Maritime University, Dalian 116026, China; 2 Houston International Institute, Dalian Maritime University, Dalian 116026, China

**Keywords:** multi-material fused filament fabrication, PLA-based composites, sandwich panels, flexural properties, Taguchi method

## Abstract

This study prepares composite panels with three Polylactic acid (PLA)-based materials via the multi-material fused filament fabrication method. The influences of four processing parameters on the mechanical properties of 3D-printed samples are investigated employing the Taguchi method. These parameters include the relative volume ratio, material printing order, filling pattern, and filling density. A “larger is better” signal-to-noise analysis is performed to identify the optimal combination of printing parameters that yield maximum bending strength and bending modulus of elasticity. The results reveal that the optimal combination of printing parameters that maximizes the bending strength involves a volume ratio of 1:1:2, a material sequence of PLA/foam-agent-modified eco-friendly PLA (ePLA-LW)/glass fiber-reinforced eco-friendly PLA (ePLA-GF), a Gyroid filling pattern, and a filling density of 80%, and the optimal combination of printing parameters for maximum bending modulus involves a volume ratio of 1:2:1 with a material sequence of PLA/ePLA-LW/ePLA-GF, a Grid filling pattern, and 80% filling density. The Taguchi prediction method is utilized to determine an optimal combination of processing parameters for achieving optimal flexural performances, and predicted outcomes are validated through related experiments. The experimental values of strength and modulus are 43.91 MPa and 1.23 GPa, respectively, both very close to the predicted values of 46.87 MPa and 1.2 GPa for strength and modulus. The Taguchi experiments indicate that the material sequence is the most crucial factor influencing the flexural strength of the composite panels. The experiment result demonstrates that the flexural strength and modulus of the first material sequence are 67.72 MPa and 1.53 GPa, while the flexural strength and modulus of the third material sequence are reduced to 27.09 MPa and 0.72 GPa, respectively, only 42% and 47% of the first material sequence. The above findings provide an important reference for improving the performance of multi-material 3D-printed products.

## 1. Introduction

Additive manufacturing (also known as 3D printing) creates complex geometries by depositing materials in a layer-by-layer manner [1]. Unlike traditional subtractive methods, Additive Manufacturing (AM) reduces material and time costs in parts and tooling rapid-prototyping [2], making it a widely used technique across various industries [3,4,5]. Among the AM approaches, material extrusion AM is one of the most popular methods due to its low cost in hardware and high ability to deal with a wide range of polymeric materials. Fused Filament Fabrication (FFF) is a common approach within material extrusion AM, where a continuous thermoplastic filament is melted via a heated nozzle and then deposited onto a pre-heating print-bed to form 3D objects. Engineering plastics, including Acrylonitrile Butadiene Styrene (ABS), Polylactic Acid (PLA), Polycarbonate (PC), and others, can be easily processed by FFF systems. Among these, PLA has garnered significant attention due to its high degree of biodegradability, which is environmentally friendly [6,7]. Nevertheless, virgin PLA polymers exhibit relatively low mechanical properties as compared to other competitive plastics products (e.g., ABS, PC) due to their linear molecular structures. To overcome this limitation, fibrous reinforcements are incorporated into the PLA matrix to produce PLA composites with superior mechanical performances [7,8,9]. Dong et al. [10] investigated the effects of coconut shell fiber content and alkali treatment on the mechanical, thermal, and biodegradable properties of PLA/coconut shell fiber bio-composites fabricated by the compression molding process. Their study found that the treated fiber composites had improved the tensile and flexural moduli. Although natural fiber-reinforced PLA materials exhibit higher environmental friendliness, natural fibers are less strong and more hydrophilic, which can accelerate the degradation of PLA materials, thereby hindering their practical application in engineering. In contrast, synthetic fibers such as carbon fibers and glass fibers [9,11] possess superior mechanical properties. Ruz-Cruz et al. [12] fabricated cellulose microfibers (MFCs)-reinforced PLA composites and a blend of MFCs and cellulose nanocrystals (CNCs)-reinforced PLA composites. The thermomechanical properties of these multi-scale PLA biocomposites were investigated using thermogravimetry (TGA), differential scanning calorimetry (DSC), bending mechanics, and dynamic mechanics tests (DMA), revealing that replacing MFCs with CNCs within the range of 1–5% effectively enhances the thermal stability of the materials, improves the crystallization of PLA materials, and increases the bending mechanical properties at room temperature by ~40%. The crystallinity of a polymer affects its physical properties, such as hardness, modulus, tensile strength, stiffness, and melting point, and for PLA, to some extent, its degradation rate [13]. Glass fibers are the most extensively used fibers for reinforcing polymers due to their excellent mechanical properties, low cost, and high heat resistance [11,14]. Chicos et al. [15] evaluated the impact of 3D printed packing density on the mechanical and thermal properties of short glass fiber-reinforced PLA specimens. Their findings indicated that the specimens exhibited optimal mechanical properties at 100% filling density, while those with 50% and 75% filling density demonstrated higher toughness compared to their 100% filling density counterparts. In addition to fiber fillers, several studies also reported that the polymer additives were effective in modifying the material properties of PLA [16,17,18,19,20]. The addition of foaming agents resulted in the production of lightweight PLA materials with high porosity and a high strength-to-weight ratio. Kanani et al. [21] prepared foamed-PLA using FFF systems tensile, where they observed that the foaming expansion was directly related to the extrusion temperature and nozzle moving speed. Damanpack et al. [22] conducted a parallel study and reported that the microscale material bonding of FFF-deposited foamed PLA materials was significantly influenced by the printing temperature.

The aforementioned observations indicate that the reinforced PLA materials exhibit enhanced mechanical performances when compared to the virgin polymer. Furthermore, in light of the recent advanced multi-material FFF systems, it is now feasible to 3D print composite panels utilizing various feedstock materials, thereby incorporating multiple functionally reinforced composites into a single entity (e.g., [23,24,25,26]). Kamaal et al. [27] aimed at the mechanical properties of 3D-printed carbon fiber-reinforced polylactic acid composite (CF-PLA) in the fused deposition modeling (FDM) method and attained the best parameter set that provides the maximum strength using the minimum material. In addition, regarding PLA-TPU (Thermoplastic polyurethanes) and PLA-ABS, Yavas et al. [28] explored the microstructural and mixed-mode fracture characteristics of the PLA-TPU interfaces and improved the energy absorption capacity. Rasheed et al. [29] utilized Taguchi orthogonal optimization to study the influence of distinct FDM process parameters on the mechanical properties, and they determined the optimal parameters for better tensile strength for a bi-layered composite of PLA-ABS. Another set of studies on PLA-ABS introduced the idea of laying down a high-toughness material on the 3D-printed carbon fiber-reinforced polymer composite sheet. Ahmed et al. [30] made a hybrid composite of laminar structures and printed the CF-PLA/ABS hybrid laminar composite. The impact toughness was optimized by adjusting the 3D printing parameters, and the fracture surface was characterized. Recently, short-carbon fiber-filled PLA and toughness-enhanced-additive-modified PLA were integrated into layered composite specimens. The resulting integrated specimens displayed a combined and adjustable tensile strength contingent on the relative fraction of the two constituents [31]. To further explore the functional complexity of the composite panels fabricated using multi-material FFF systems, composite sandwich panels composed of three types of PLA-based ingredients are prepared, including virgin PLA, rigid glass fiber reinforced PLA (PLA-GF), and foam-agent-modified PLA (also referred to as lightweight PLA, i.e., PLA-LW). The effects of various processing parameters on the composite panel preparation are evaluated, such as the constituent material stacking-order, relative fraction, printing infill pattern, and infill density. The Design of Experiments The taguchi method is employed to reduce the extensive number of experiments. The flexural properties of the specimens are utilized as representatives of material performance. By comparing the measured data, the optimal processing parameters for achieving superior material properties are recommended.

The presented study highlights that the sequence of material printing is a crucial determinant of the bending performance of sandwich specimens, emphasizing its significance for future studies of multi-material 3D printing. Regarding the scope of this study, the manufacturing approach offers a novel perspective for the design and production of protective materials. These sandwich panels can be employed in wearable protective equipment such as helmets, sports knee pads, and medical braces. Our study also fills the gap in the current research on the preparation parameters of sandwich composite panels made of PLA-based composites.

## 2. Materials and Methods

The multi-material FFF system applied for the specimen fabrication is also presented. Furthermore, the procedure of our Design of Experiment (DOE) with the Taguchi method is discussed.

### 2.1. Materials

**Figure 1:**
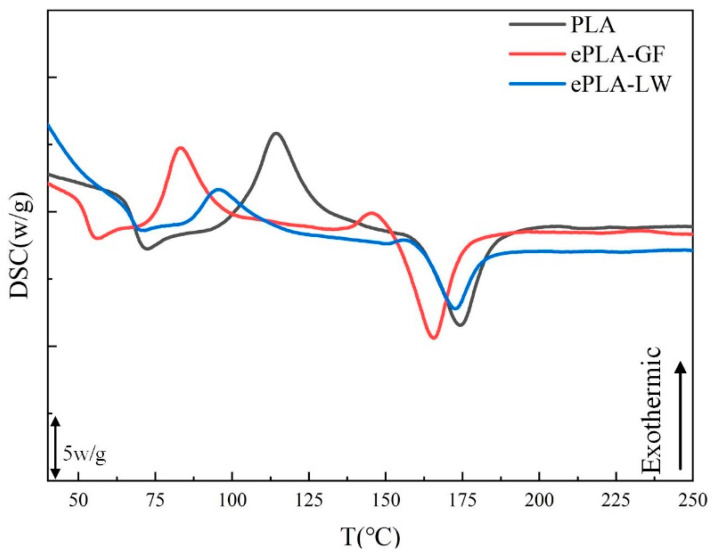
Three materials: DSC Curves.

In this study, PLA and its composite materials, ePLA-LW and ePLA-GF, are employed (note that ePLA refers to an eco-friendly PLA resin that is designed to enhance the bio-degradabilities of PLA composites). The feedstock filaments are supplied by Esun (Shenzhen Esun Industrial Co., Ltd., Shenzhen, China). As the selected materials are PLA-based, we would expect that the multi-material 3D-printed specimens exhibit a high quality of interlayer adhesion. Therefore, in the results discussion, we could ignore the effect of interlayer bonding issues. Finally, the material properties provided by the supplier are given in Table 1. Differential scanning calorimetry (DSC-500C.Jiezhun Instrument Equipment Co., Ltd., Shanghai, China) is employed to test three materials, and the DSC curves for these materials are presented in Figure 1. Compared to pure PLA, the glass transition temperature, cold crystallization temperature, and melting temperature of ePLA-GF have all decreased. The cold crystallization and melting peaks of the ePLA-LW material also diminish. This study indicates that the crystallinity of PLA material is influenced by chemical modification during the foaming process [32]. This may be attributed to the addition of the foaming agent, which hinders the development of the crystalline structure in PLA.

Notably, ePLA-GF filament is reinforced with 16 wt.% short glass fiber, resulting in enhanced stiffness and strength. ePLA-LW filament is modified by adding a thermally sensitive foaming agent, i.e., allowing for increased porosity with elevated nozzle temperatures (the recommended temperature range is from 210 to 270 °C). From pre-experiments on the printing temperature of ePLA-LW, it was found that the temperature of 230 °C with an extrusion rate of 75% exhibits the best expected performance. The detailed results from these preliminary experiments are provided in Appendix B for reference. The resulting foamed PLA exhibits lower material density and rigidity, but higher toughness. Finally, a multi-material FFF system is employed, utilizing the constituent materials in a prescribed layer-stacking formation. It is anticipated that the designed composite panel will exhibit favorable material properties.

### 2.2. Three-Point Bending Specimen Preparation with Multi-Mat FFF

The three-point bending test specimens are designed in accordance with the ASTM D790 standard [33]. SolidWorks 2020 (Dassault Systèmes Corp., Vélizy-Villacoublay, France) is used for generating the STL digital file. Cura 4.13.0 (Ultimaker Corp., Utrecht, The Netherlands) slicing software is employed to configure the printing parameters and generate the corresponding G-code file for the fabrication process. As previously mentioned, three distinct constituent materials are incorporated within the specimen. Given the layer-by-layer manufacturing nature of FFF, the specimen is ultimately fabricated as a sandwich panel. A GEEETECH A10T (Shenzhen Geeetech Technology Co., Ltd., Shenzhen, China) 3-in/1-out multi-material FFF 3D printer is utilized to print sandwich specimens. The 3D printer featured three extruders, enabling control over up to three material filaments extruded from a single nozzle. It should be noted that the original G-code file generated in Cura 4.13.0 does not guarantee successful printing of the sandwich structure composed of three materials by the GEEETECH A10T. To address this issue, the number of layers, printing temperature, and extrusion rate for each material are modified in the code according to the printing parameters of the three materials and then imported into the 3D printer for printing.

To properly establish the printing parameters of the constituent materials, pre-experiments are conducted. To ensure optimal conditions for the fabrication of the bending sandwich specimen, the filaments are uniformly dried at 50 °C for 5 h. This pre-drying process helps establish an ideal condition for the filaments prior to their use in the printing process. A series of pre-tests are performed on specimens with varying parameters, specifically noting that the heat-foaming ePLA-LW exhibits the best mechanical properties prior to foaming (i.e., nozzle temperature at ~210 °C). Conversely, the density of ePLA-LW decreases as the degree of foaming increases. In our case, the nozzle temperature of the ePLA-LW is set at 230 °C with an extrusion rate of 75%, resulting in a relatively high strength-to-weight ratio property. The general printing parameters are finalized as appearing in Table 2, aiming to achieve consistent and superior flexural strength.

### 2.3. Design of Experiments

**Figure 2:**
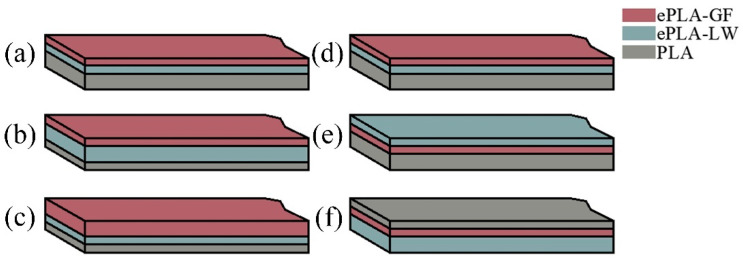
Diagram of Volume Ratio: (**a**) 2:1:1, (**b**) 1:2:1, (**c**) 1:1:2; The schematic diagram refers to the ordering of materials based on a sample volume ratio of 2:1:1. (**d**) PLA/ePLA-LW/ePLA-GF; (**e**) PLA/ePLA-GF/ePLA-LW; (**f**) PLA/ePLA-LW/ePLA-GF/PLA.

**Figure 3:**
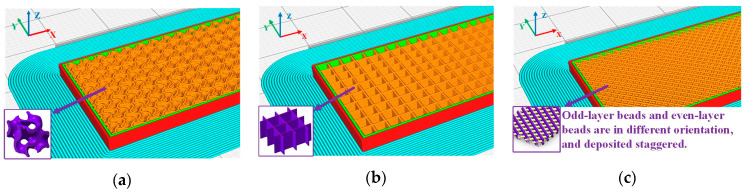
Filling pattern when filling density is 40%: (**a**) Gyroid, (**b**) Grid, (**c**) 45°/135° Lines (Different colors indicate the overlap of 45°/135° straight lines between layers).

**Figure 4:**
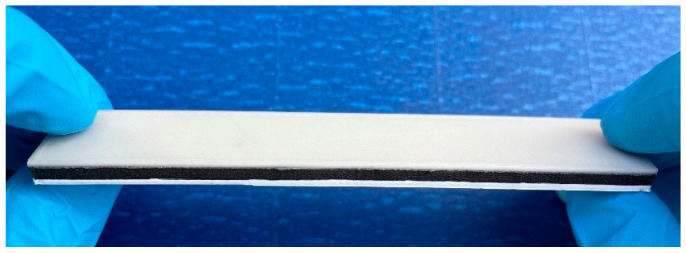
Example of the printed sandwich specimen (volume fraction ratio 1:2:1, material stacking order: PLA/ePLA-LW/ePLA-GF).

The influence of four essential processing parameters on the flexural properties of printed composite panels is examined in this study. These parameters include constituent material volume fraction ratio, material stacking order, infill pattern, and filling density. For open-ended FFF 3D printing, previous studies often employed the Design of Experiments (DoE) Taguchi method to optimize the printing parameters when dealing with varying materials (e.g., [34,35,36,37,38,39]). Consequently, the Taguchi method was also employed to efficiently perform the experimental tests. Accordingly, a L_9_ orthogonal array was generated to test the effects of the four parameters at three levels, reducing the originally large number of tests to nine trials in total [35]. As shown in Table 3, we established three-level values for each parameter factor. The volume ratio indicated the volume of each material in the sandwich specimen, which is divided into four equal parts according to the deposition direction and then partitioned into three sections in the form of Figure 2a–c. The material stacking order refers to the sequence of the three materials in the deposition direction, and in this study, three distinct orders are adopted, as demonstrated in Figure 2d–f. The printing pattern employed in this study corresponds to the internal filling structure pattern within the specimen. Each sandwich specimen is filled with a specific infill pattern throughout the entire sample. Within Cura 4.13.0 software, three distinct filling patterns are chosen: Gyroid, Grid, and 45°/135° Lines (as depicted in Figure 3). Additionally, a brim configuration is implemented in Cura 4.13.0 (highlighted in the blue section of Figure 3) to enhance material adhesion to the printing platform. Note that the 45°/135° Lines filling pattern represents an overlap of lines between different layers, while the Grid filling pattern involves an overlap within the same layer. To preclude material intrusion caused by specimen deformation, top-bottom layers between different materials are incorporated to separate them. This design increases the contact area and facilitates intermolecular diffusion, thereby bolstering interlayer bonding strength and effectively ameliorating the mechanical properties of the specimen. Furthermore, as illustrated in Figure 4, a printed sandwich specimen example is demonstrated where all three materials are employed [25].

## 3. Results

The Taguchi method was employed in this study to efficiently identify the optimal combination of processing parameters by reducing the number of trials. Initially, ANOVA analyses were performed on our measured flexural properties. The results revealed that all the investigated parameters (cf. Table 3) highly contribute to the experimental results in a similar way, making the differences between the parameters indistinguishable (to be concise, the ANOVA results were provided in the Appendix A). Alternatively, the Taguchi method categorizes the experimental factors into controllable and noisy components. Controllable factors are parameters or variables that can be controlled and remain constant following selection. Noisy factors refer to variables that are uncontrollable under normal conditions, i.e., random variables. The Signal-to-Noise ratio (S/N) is the resistance to noise interference. A higher S/N ratio indicates a control factor setting that minimizes the impact of the noise factor, resulting in the specimen having more consistent and stable performance. The S/N ratio quantifies the effect of multiple parameters on the experimental output values by calculating the signal-to-noise value to identify the optimal parameter settings [40]. In Taguchi experiments, there are three types of expectations: expecting small (smaller is better), expecting large (larger is better), and expecting eye type (nominal is better) [38]. Herein, the “larger is better” analysis for optimization of stiffness and strength is adopted. The equations for “larger is better” can be written as

(1) $$S/N=-10\log\left[\frac{1}{n}\sum_{i=1}^{n}\frac{1}{y_{i}^{2}}\right]$$

where $y_{i}$ refers to the experimental results of the i-th test, and *n* is the total number of the tests. Herein, five specimens are prepared for each experimental group to undergo bending testing. The mean value of the orthogonal experiment test is derived by eliminating the maximum and minimum values from the results of each experimental group, and thus n = 1. Additionally, taking the average of the other three specimens as the experimental values, the experimentally measured flexural properties of the FFF-produced sandwich specimens and the S/N analysis are presented.

### 3.1. S/N Analyses on Measured Flexural Properties

**Figure 5:**
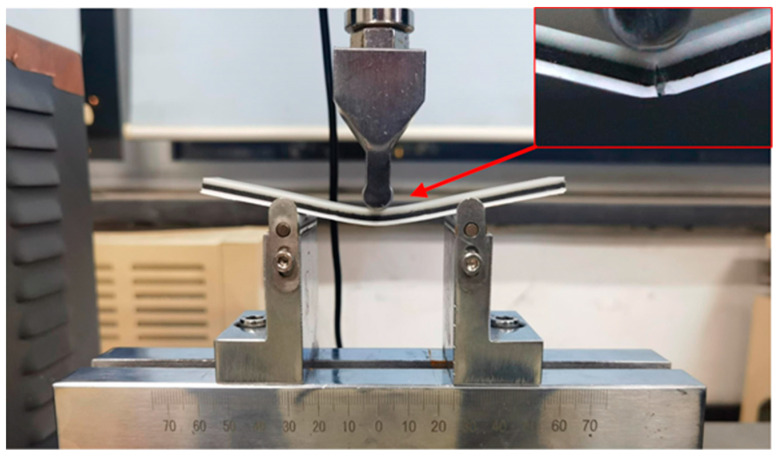
Three-point bending test.

**Figure 6:**
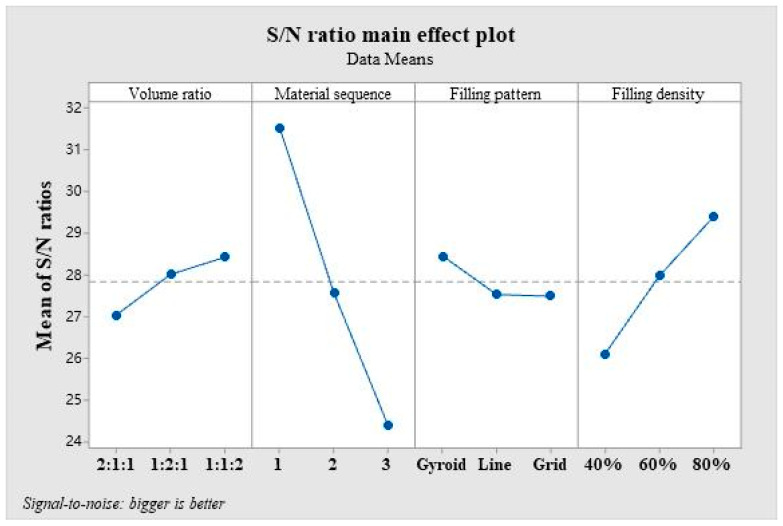
Main effects plot of the levels of each factor on the bending strength.

According to the orthogonal experimental sequence presented in Table 3, each group of experimental samples undergoes a three-point bending test utilizing a GTM universal material testing machine capable of exerting a maximum force of 10 KN (Xie-Qiang Instruments Manufacturer Corp., Shanghai, China). The bending span measures 50 mm, and a loading speed of 2 mm/min is applied, as illustrated in Figure 5 The bending strength is analyzed employing the “larger is better” signal-to-noise ratio analysis in Minitab 19 (Minitab, LLC, State College, PA, USA). The experimental values and signal-to-noise ratio values are provided in Table 4. The average value of the experimental results of the three samples within each group is computed. Table 5 displays the signal-to-noise ratio values at various levels of each parameter. Delta signifies the range of signal-to-noise ratios at different levels of the same factor. The width of the range indicates the extent of impact distinct levels of alteration have on the empirical outcomes. Considering the size of the range is crucial in determining the level of impact, as a broader range indicates a greater degree of influence. Consequently, evaluating the range size is crucial when determining the magnitude of change required to achieve a significant effect. Referring to the data presented in Table 5, it is evident that the material sequence yields the most pronounced influence on the bending strength, whose delta reaches 7.15, followed by the filling density with a delta of 3.29. In contrast, the filling pattern exhibits the least impact on the bending strength among the processing parameters evaluated in this study, with the smallest delta of 0.96. Drawing conclusions from the main effect plots of the signal-to-noise ratio as depicted in Figure 6, the combination of processing parameters leading to the maximum bending strength consists of a material volume ratio of 1:1:2, a material sequence of PLA/ePLA-LW/ePLA-GF, a Gyroid filling pattern, and a filling density of 80%.

**Figure 7:**
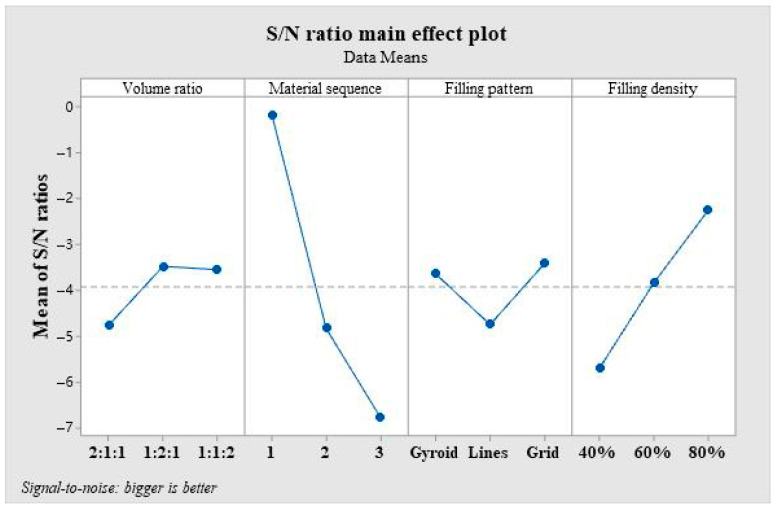
The main effects plot shows the levels of each factor on the bending modulus.

Moreover, the signal-to-noise ratio analysis is conducted on the flexural modulus of the composite penal samples. Table 6 shows the nine experimental results and signal-to-noise ratio values listed in the orthogonal experiment. In Table 7, we further provide the response values, where the degree of influence of different levels between individual factors on the flexural modulus can be clearly seen. The largest range of signal-to-noise ratios was observed under the factor of material sequence at three levels, up to 6.59. This indicates variations in material sequence can significantly affect the bulk stiffness of the composite panels. It is noted that the material sequence is a crucial factor affecting bending strength, followed by filling density (The range is 3.44). However, unlike the trend of strength data, the volume ratio demonstrated minimal impact on the results of elastic modulus, whose delta are only 1.28. Overall, our findings indicate that the material sequence parameter exerts the most significant influence on both the strength and stiffness of samples in this experimental model. Furthermore, filling density also significantly impacts the flexural performance of samples. By analyzing the signal-to-noise ratio at each level, an optimal combination can be identified via Figure 7, which maximizes the stiffness of the samples. Ultimately, a favorable set of processing parameters is suggested as follows: The volume ratio is 1:2:1, the material order is PLA/ePLA-LW/ePLA-GF, the fill pattern is Grid, and the fill density is 80%.

**Figure 8:**
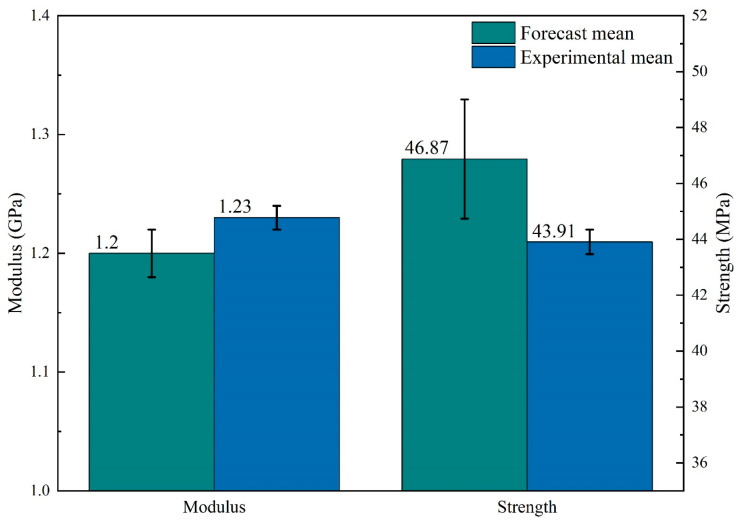
Taguchi comparison of predicted values and experimental values.

Furthermore, optimizing the combination of processing parameters beyond the selected levels, as previously studied, can lead to enhanced flexural strength and modulus properties, as predicted by the Taguchi method. We validate the predicted values through relevant experiments, as illustrated in Figure 8. In each experiment, three-panel samples are prepared to calculate the mean value and standard deviation of the measured properties. The small values of the standard deviation indicate the high stability of the experimental data. By comparing the validation experimental measurements with the predicted values (as in [39,41]), it is observed that this experimental model aligns with the prediction of the Taguchi method. The experimental values are 1.23 GPa and 43.91 MPa for modulus and strength, which approximate the predicted values of 1.2 GPa and 46.87 MPa, respectively. The Taguchi method can predict an additional 72 experimental data points outside of the 9 groups of experiments presented in the orthogonal table of this paper, thereby expanding the optimization area of the processing parameters.

### 3.2. Meso-Structural Analysis of Failure Mechanisms

**Figure 9:**
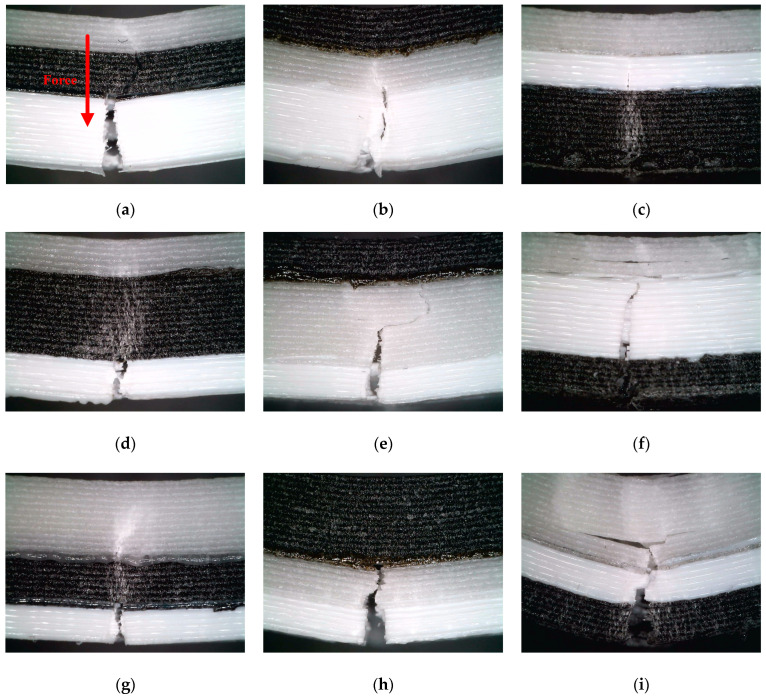
Crack propagation from a side view (The loading direction for all specimens in the figures is consistent with (**a**)). Note that numbers 1–9 correspond to the experimental cases appearing in Table 3. (**a**) Number 1; (**b**) Number 2; (**c**) Number 3; (**d**) Number 4; (**e**) Number 5; (**f**) Number 6; (**g**) Number 7; (**h**) Number 8; (**i**) Number 9.

**Figure 10:**
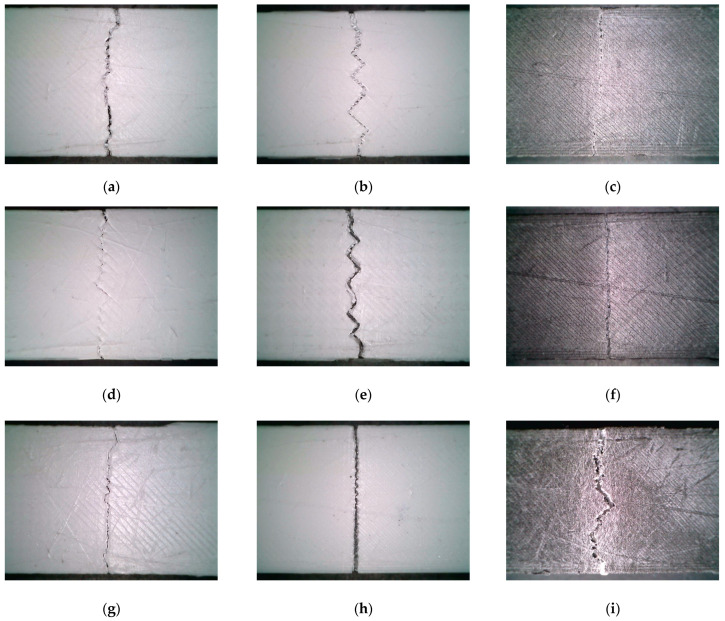
Crack propagation from a bird’s-eye view. Note that numbers 1–9 correspond to the experimental cases appearing in Table 3. (**a**) Number 1; (**b**) Number 2; (**c**) Number 3; (**d**) Number 4; (**e**) Number 5; (**f**) Number 6; (**g**) Number 7; (**h**) Number 8; (**i**) Number 9.

**Figure 11:**
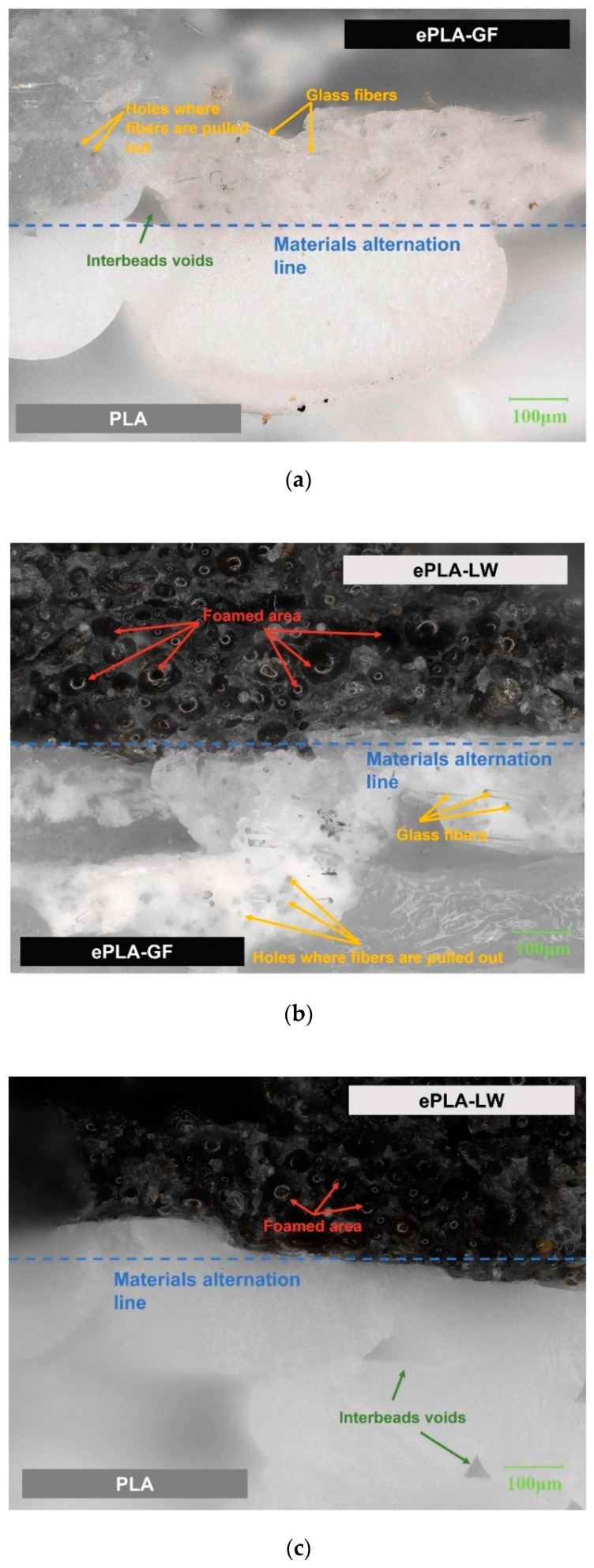
Microscopic imaging of interfaces between different materials: (**a**) PLA and ePLA-GF; (**b**) ePLA-LW and ePLA-GF; (**c**) PLA and ePLA-LW.

Based on the results of Section 3.1, we further analyzed the fracture mechanism of the sandwich structure under bending load. Using the Dino-lite (AnMo Electronics Corp., Taiwan, China) handheld microscope, the morphology of a single sample crack in each group of experiments in Table 3 is observed from the Side view and Bird view perspectives, as shown in Figure 9 and Figure 10. The Keyence VHX-7000 optical microscope was employed to observe the material interfaces between PLA and ePLA-GF, PLA and ePLA-LW, as well as ePLA-GF and ePLA-LW, as shown in Figure 11.

The infill density determines the amount of material present in the printed part, which directly affects its strength and stiffness. Higher infill density generally leads to higher strength, as there are more material layers and interconnections between them. Moreover, the arrangement and orientation of the printed material are determined by the infill pattern. The chosen infill pattern can influence interlayer adhesion and bonding, thereby impacting the overall mechanical performance of the printed part. Consequently, adjusting both infill density and pattern becomes crucial as they directly affect material distribution within the printed object, subsequently influencing its mechanical characteristics such as strength and stiffness [42]. We can assess the mechanical properties and further adjust the infill density and pattern based on crack propagation in the sample.

By examining the crack propagations of samples from the side view (cf. Figure 9), it is observed that fracture cracks predominantly initiate and propagate in the PLA and ePLA-GF layers. The ePLA-LW layer, however, demonstrates a lower likelihood of failure due to its high toughness. Consequently, an increased relative volume of the ePLA-LW layer can decrease the likelihood of crack propagation (e.g., Figure 9d). Alternatively, the ePLA-GF layer exhibits a high probability of fracture, often characterized by delamination within the inter-beads of the layer, causing the crack to extend laterally along the inter-bead boards (e.g., Figure 9f). Furthermore, the crack expansion between inter-beads within the PLA layer is relatively parallel to the direction of the applied bending force. However, the PLA layer demonstrates a relatively low capacity for resisting cracks once the crack initiates (e.g., Figure 9a). Figure 10 offers a bridged view of the crack propagations (i.e., of the outermost layer of the samples’ bottom surface). The filling pattern and filling density have a significant impact on the crack. Under the same filling density, samples with a 45°/135° Lines filling pattern exhibit a zigzag line at the bottom of their cracks, leading to irregular serrations (taking 60% filling density as an example, such as Figure 10b,f,g), while Gyroid-filled samples display a relatively straight line of bottom cracks. Notably, although different filling patterns are employed, the shape of the crack tends to be straighter as the sample filling density increases. Furthermore, the smaller the density, the higher the degree of zigzag in the resulting bottom crack, suggesting that the filling pattern predominantly governs the crack line formation at relatively low infill density, with the infill density becoming a more dominant factor as it increases to a larger value.

Furthermore, the characterization of the microstructures depicted in Figure 11 reveals that the interfacial adhesion between each pair of the constituent materials is in excellent condition, which may be likely attributed to our pre-designed top-bottom layers for each sub-part of the sandwich material, i.e., the outermost side of each material layer is printed with fully saturated materials. This configuration also enhances the interlayer bonding quality between distinct materials. Given these configurations, it is postulated that the interlayer adhesions between different constituent materials should not detract from the bulk mechanical performance of the 3D-printed sandwiches when subject to external bending loads. However, it is noted that the impact of interlayer bonding quality is a crucial factor in determining the material properties of the sandwich samples under other loading conditions. Future in-depth studies are desired to further explore these aspects.

### 3.3. Controlled Variables Method on the Material Stacking Order

**Figure 12:**
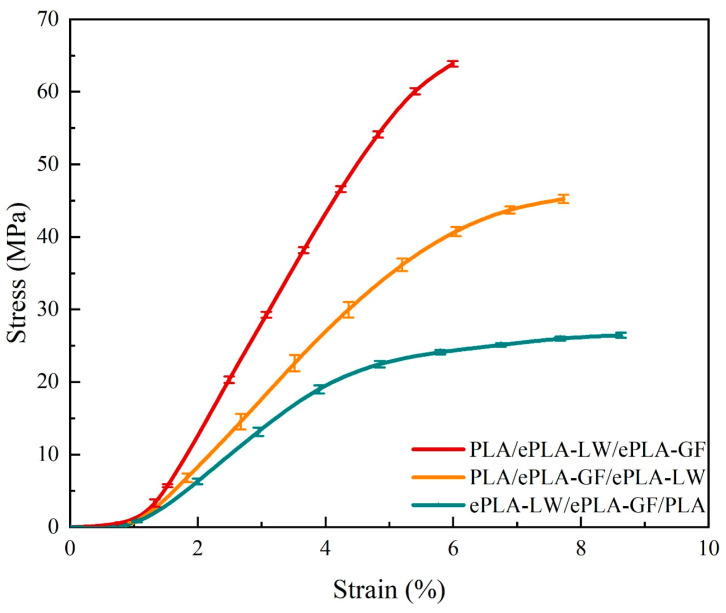
Three-point bending mechanical curve.

It should be highlighted that the interactive effects among the four investigated parameters on the printed components have been disregarded, assuming that each processing parameter produces independent effects on the resulting material properties. From the experimental results, it is evident that the arrangement of materials has a significant influence on the flexural strength and modulus of composite panels fabricated using multi-material FFF. To further delve into this factor, an additional study under the controlled variables method is conducted to assess the impact of material sequence on the strength and modulus of printed samples. The printing parameters for the samples are specified as follows: 45°/135° Line filling pattern and 100% filling density, with other general conditions identical to those outlined in Table 2. The samples in this controlled variables method utilize the same ASTM-D790 standard, except for a slight modification in thickness to 5.4 mm, resulting in equal-length pieces for the three layers of constituent materials. Note: Figure 2d–f depicts the three levels employed in this experiment. Figure 12 exhibits the stage curves of three groups of samples during three-point bending tests [38] (note that the curves are terminated at the maximum bending strength). We combined three samples at each level into one curve and depicted the discrepancy between different sample curves through error bars. The results indicate that the first-level sample exhibits both maximum stiffness and strength, while the plastic deformation area of the third-level sample is larger than its elastic deformation area, suggesting superior ductility. The stiffness of the second-level sample surpasses that of the third-level sample, yet its ductility is better than that of the first-level sample.

**Figure 13:**
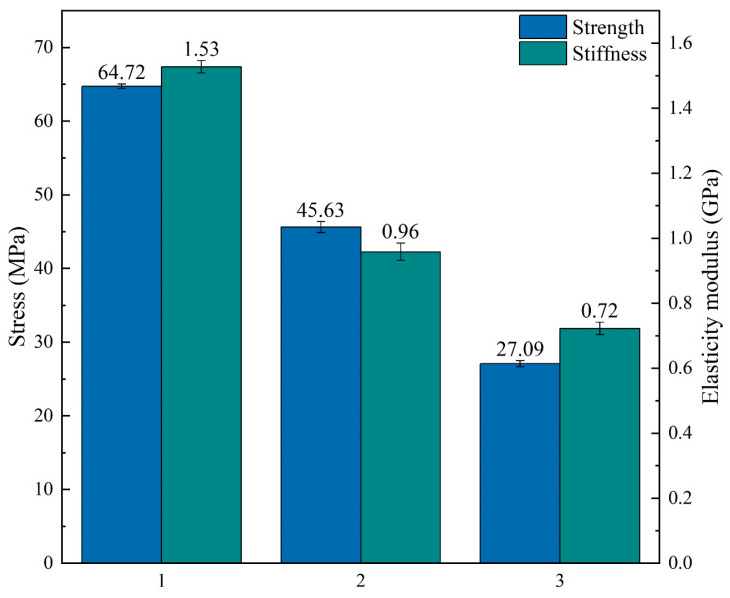
Histogram of average mechanical data for each horizontal bend.

Furthermore, we compute the mean values of the maximum strength and modulus in the three experimental groups, along with the error bars of the data, as depicted in Figure 13. The results indicate that the first level of material order exhibits the highest strength and stiffness. Samples prepared using the “PLA/ePLA-LW/ePLA-GF” sequence exhibited smaller error bars, suggesting more stable mechanical properties. As illustrated in Figure 13, the first level demonstrates the highest bending strength, reaching 64.72 MPa, while the third level exhibits the lowest bending strength (only 27.09 MPa), at ~42% of the bending strength of the first level samples. The second level exhibits a higher bending strength than the third level, up to 45.63 MPa, at ~71% of the first level. However, the mechanical properties of the second-level sample are less stable than those of the other two levels, as indicated by the error bar. In addition, Figure 13 depicts the maximum flexural modulus of the three levels of samples. The first level sample exhibits the highest modulus at 1.53 GPa, followed by the second sample, which decreases to 0.96 GPa at only ~63% of the first level sample. The error bar of the second level sample is the largest, while the third level exhibits the lowest modulus of 0.72 GPa at ~47% of the first level sample.

Under the controlled variables method, our data reveal that the distinct printing sequences of the three materials have a substantial impact on the mechanical properties of the samples, with the maximum disparities in bending strength and stiffness among different stacking orders being approximately 58% and 53%, respectively. It is worth noting that the interface adhesion quality among the constituent materials could significantly contribute to determining the bulk flexural properties of the printed panels (e.g., [43]). Consequently, the material stacking order is deemed the most critical processing parameter in the 3D printing preparation of the composite panels. It is important to highlight that the interlayer bonding strength between different materials is intricately associated with several factors, such as thermodynamics [23], chemical affinity [44], and FFF process parameters [45]. It is anticipated that complexity will be delved into further through separate, in-depth studies.

## 4. Conclusions

Utilizing the Taguchi method, we design an orthogonal experiment designed to investigate the impact of four processing parameters on the mechanical properties of the prepared samples, namely volume ratio, material printing sequence, filling pattern, and filling density.

By employing a larger-is-better signal-to-noise ratio analysis, we identify the optimal parameter combinations for achieving maximum flexural strength and stiffness properties. The combination that maximizes flexural strength includes a volume ratio of 1:1:2, a material sequence of PLA/ePLA-LW/ePLA-GF, a Gyroid filling pattern, and an 80% filling density. Similarly, the combination that maximizes flexural modulus consists of a volume ratio of 1:2:1, a material sequence of PLA/ePLA-LW/ePLA-GF, a Grid filling pattern, and 80% filling density. They have the largest S/N ratios of 7.15 and 6.59 compared to other combinations.

The Taguchi prediction method is applied to forecast the performance of an optimized processing parameter combination obtained from signal-to-noise ratio analyses. Subsequently, the predicted results are validated through related experiments, demonstrating the accuracy of the Taguchi prediction with a minimal deviation between experimental and true values of less than 10%. The experimental values of modulus and strength are 1.23 GPa and 43.91 MPa, which are more or less the same as the predicted values of 1.2 GPa and 46.87 MPa, respectively.

Through the analysis of signal-to-noise ratios across various processing parameter levels, we discover that the material stacking order (the range reaching 6.59) is the most significant factor influencing the flexural performance of the PLA composite panels. Consequently, we conducted a controlled variables method experiment as applied to the three levels of the material stacking order parameter, revealing that the first material sequence exhibits the highest flexural strength and modulus with the smallest error bar, at 67.72 MPa for strength and 1.53 GPa for modulus. In contrast, the third material sequence demonstrates the lowest strength and stiffness while displaying higher ductility in mechanical performance. Its flexural strength and modulus, declining to 27.09 MPa and 0.72 GPa, respectively, are 42% and 47%, respectively, compared to the first material sequence.

This research concluded that the Taguchi method is a useful tool for optimizing printing parameters and highlighted the importance of material stacking sequence in determining the bending performance of multi-material 3D-printed components. By adjusting the mechanical properties, printed components can be customized to suit specific applications. The manufacturing approach employed in this study introduces a novel concept for designing and fabricating protective materials with potential applications in wearable protective equipment such as helmets, sports knee pads, and medical guards.

## Figures and Tables

**Table 1:** Material properties of constituent materials from the supplier.

Material	PLA	ePLA-LW	ePLA-GF
Density (g/cm^3^)	1.2	1.2	1.31
Tensile Strength (MPa)	72	32.2	59.27
Flexural Strength (MPa)	90	41.31	85
Elongation at Break	11.8	68.9	7.99
Flexural Modulus (MPa)	1915	1701	4414.89
Heat Distortion Temp. (°C)	53	53	56
Melt Flow Index (g/10 min)	3.5 (190 °C/2.16 kg)	8.1 (190 °C/2.16 kg)	6.36 (190 °C/2.16 kg)

**Table 2:** General printing parameters are applied for specimen preparation.

Material	Nozzle Temp. (°C)	Relative Feeding Rate (%)	Number of Outline Shell	Layer Thickness (mm)	Printing Speed (mm/s)	Print-Bed Temp. (°C)
PLA	210	100	2	0.2	50	60
ePLA-LW	230	75	2	0.2	50	60
ePLA-GF	230	100	2	0.2	50	60

**Table 3:** Design of experiments with an L9 orthogonal array.

Exp. Number	Volume Ratio	Material Sequence	Filling Pattern	Filling Density
1	2:1:1	1	Gyroid	40%
2	2:1:1	2	Lines	60%
3	2:1:1	3	Grid	80%
4	1:2:1	1	Lines	80%
5	1:2:1	2	Grid	40%
6	1:2:1	3	Gyroid	60%
7	1:1:2	1	Grid	60%
8	1:1:2	2	Gyroid	80%
9	1:1:2	3	Lines	40%

**Table 4:** Bending strength and S/N ratio values.

Number	Volume Ratio	Material Sequence	Filling Pattern	Filling Density	Bending Strength (MPa)	Standard Deviation	S/N (dB)
1	2:1:1	1	Gyroid	40%	30.44	0.45	29.67
2	2:1:1	2	Lines	60%	21.49	0.14	26.65
3	2:1:1	3	Grid	80%	17.47	0.35	24.84
4	1:2:1	1	Lines	80%	44.73	0.39	33.01
5	1:2:1	2	Grid	40%	19.31	0.29	25.71
6	1:2:1	3	Gyroid	60%	18.58	0.93	25.36
7	1:1:2	1	Grid	60%	39.65	0.15	31.96
8	1:1:2	2	Gyroid	80%	33.30	2.93	30.38
9	1:1:2	3	Lines	40%	14.09	0.16	22.98

**Table 5:** The S/N ratio response value to bending strength.

Level	Volume Ratio	Material Sequence	Filling Pattern	Filling Density
1	27.05	31.55	28.47	26.12
2	28.03	27.58	27.54	27.99
3	28.44	24.39	27.51	29.41
Delta	1.39	7.15	0.96	3.29
Ranking	3	1	4	2

**Table 6:** Bending modulus and S/N ratio values.

Number	Volume Ratio	Material Sequence	Filling Pattern	Filling Density	Flexural Modulus (AVG) (GPa)	Standard Deviation (×10^−2^)	S/N (dB)
1	2:1:1	1	Gyroid	40%	0.75	0.37	−2.48
2	2:1:1	2	Lines	60%	0.48	0.63	−6.38
3	2:1:1	3	Grid	80%	0.54	0.58	−5.40
4	1:2:1	1	Lines	80%	1.14	2.40	1.13
5	1:2:1	2	Grid	40%	0.52	1.13	−5.62
6	1:2:1	3	Gyroid	60%	0.51	0.09	−5.93
7	1:1:2	1	Grid	60%	1.10	1.02	0.81
8	1:1:2	2	Gyroid	80%	0.75	1.72	−2.49
9	1:1:2	3	Lines	40%	0.36	0.42	−8.97

**Table 7:** The S/N ratio response value to bending modulus.

Level	Volume Ratio	Material Sequence	Filling Pattern	Filling Density
1	−4.75	−0.18	−3.63	−5.69
2	−3.47	−4.83	−4.74	−3.83
3	−3.55	−6.77	−3.41	−2.25
Delta	1.28	6.59	1.34	3.44
Ranking	4	1	3	2

**Figure A1:**
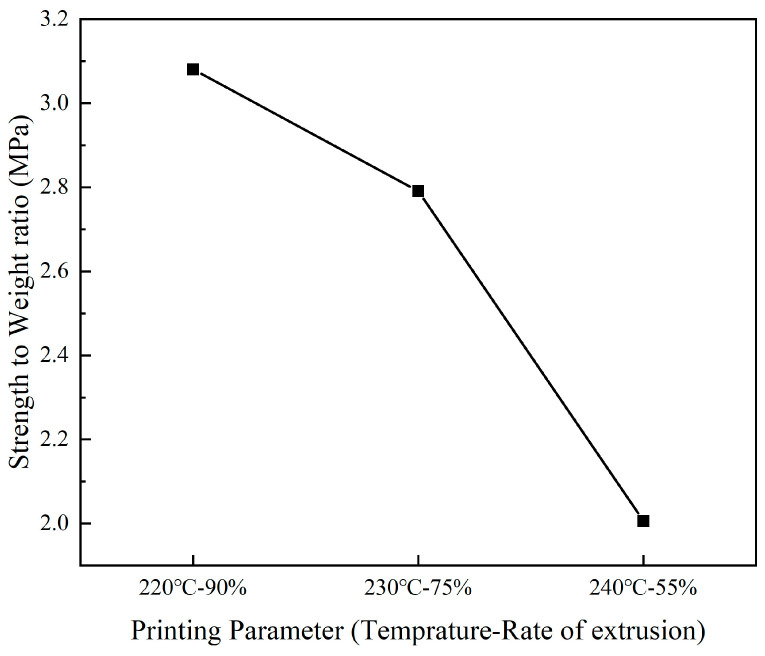
Strength-to-Weight ratio.

**Figure A2:**
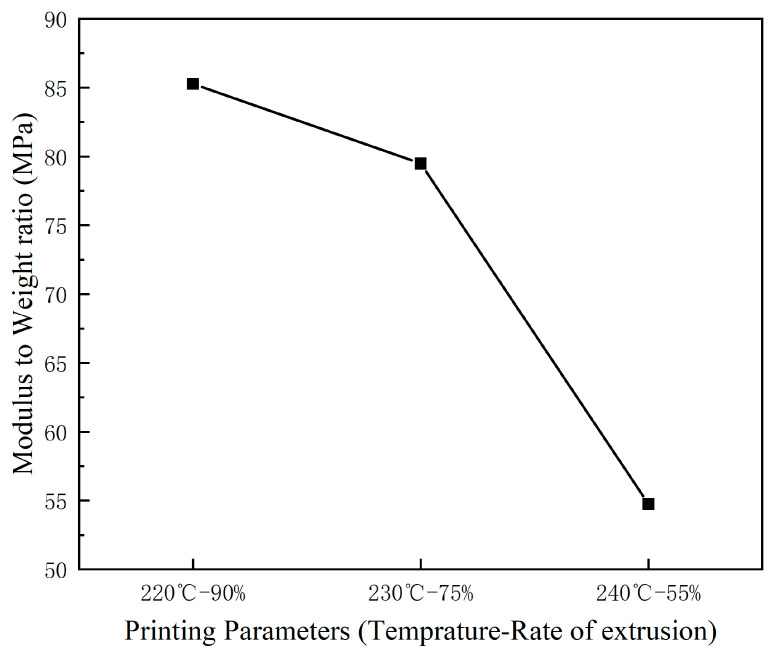
Modulus-to-Weight ratio.

## Data Availability

Data are contained within the article.

## Appendix A

The ANOVA analyses on the measured flexural strength and modulus data are performed, and the results are given in Table A1 and Table A2 as follows.

**Table A1:** Analysis of the variance of strength.

Source	DOF	SS	MS	F	P
Volume ratio	2	168.29	84.14	75.33	0.000
Material sequence	2	2137.91	1068.95	956.97	0.000
Filling pattern	2	17.92	8.96	8.02	0.003
Filling density	2	501.21	250.61	224.35	0.000
Error	18	20.11	1.12		
Total	26	2845.44			

**Table A2:** Analysis of the variance of modulus.

Source	DOF	SS	MS	F	P
Volume ratio	2	0.12	0.06	437.53	0.000
Material sequence	2	1.39	0.70	5184.48	0.000
Filling pattern	2	0.02	0.01	71.10	0.000
Filling density	2	0.32	0.16	1186.47	0.000
Error	18	0.00	0.00		
Total	26	1.85			

## Appendix B

Considering the limitations of the existing equipment in handling high temperatures, preliminary experiments are carried out according to the extrusion parameters provided by the material supplier. In order to avoid the temperature fluctuation of the nozzle caused by high-temperature printing, the test temperatures were selected at 220 °C, 230 °C, and 240 °C. For each temperature condition, we print three groups of samples and perform three-point bending experiments after obtaining the weight of each sample. We average the bending strength, bending modulus, and weight of each group of specimens as reference values. Then, we divide the average value of flexural strength and modulus by the average weight to obtain the performance-weight ratio for each group of specimens. In the following, Figure A1 represents the strength-to-mass ratio of the specimens prepared with the three printing parameters, and Figure A2 illustrates the modulus-to-mass ratio. When the printing temperature is 220 °C, the foaming effect of this sample is very poor, and there is almost no foaming. As a result, its sample performance is largely superior to other samples that have been foamed, which also leads to its higher performance-to-weight ratio. When the temperature is above 230 °C, the sample appears to be in an obvious foaming state. When the material extrusion parameters are 230 °C and the extrusion rate is 75%, the foaming effect is the best, and the performance-to-mass ratio is much higher than that of the sample printed at 240 °C.
